# Efficacy of avalglucosidase alfa on forced vital capacity percent predicted in treatment-naïve patients with late-onset Pompe disease: A pooled analysis of clinical trials

**DOI:** 10.1016/j.ymgmr.2024.101109

**Published:** 2024-06-26

**Authors:** Tahseen Mozaffar, Lionel Riou França, Jérôme Msihid, Pragya Shukla, Irina Proskorovsky, Tianyue Zhou, Magali Periquet, Kristina An Haack, Laurence Pollissard, Volker Straub

**Affiliations:** a Division of Neuromuscular Disorders, Department of Neurology, University of California, Irvine, CA, United States; b Sanofi, Gentilly, France; c Evidera, St-Laurent, QC, Canada; d Sanofi, Cambridge, MA, United States,; e Sanofi, Berlin, Germany; f John Walton Muscular Dystrophy Research Centre, Newcastle University and Newcastle Hospitals NHS Foundation Trust, Newcastle Upon Tyne, United Kingdom

**Keywords:** Late-onset Pompe disease, Enzyme replacement therapy, Avalglucosidase alfa, Alglucosidase alfa, Respiratory

## Abstract

**Background:**

The efficacy of avalglucosidase alfa (AVA) versus alglucosidase alfa (ALG) on forced vital capacity percent predicted (FVCpp) in patients with late-onset Pompe disease (LOPD) has been assessed in the Phase 3 COMET trial (NCT02782741). Due to the rarity of LOPD and thus small sample size in COMET, additional data were analyzed to gain further insights into the efficacy of AVA versus ALG.

**Methods:**

Data from treatment-naive patients with LOPD were pooled from COMET and Phase 1/2 NEO1/NEO-EXT (NCT01898364/NCT02032524) trials for patients treated with AVA, and Phase 3 LOTS trial (NCT00158600) for patients treated with ALG. Regression analyses using mixed models with repeated measures consistent with those pre-specified in COMET were performed post-hoc. Analyses were adjusted for trials and differences in baseline characteristics. Four models were developed: Model 1 considered all trials; Model 2 included Phase 3 trials; Model 3 included Phase 3 trials and was adjusted for baseline ventilation use; Model 4 included COMET and NEO1/NEO-EXT (i.e., AVA trials only).

**Results:**

Overall, 100 randomized patients from COMET (AVA, *n* = 51, ALG, *n* = 49), 60 from LOTS (ALG arm only), and three patients from NEO1/NEO-EXT (who received open-label AVA only) were considered for analysis. Mean age at enrollment was similar across trials (45.3–50.3 years); however, patients from LOTS had a longer mean duration of disease versus COMET and NEO1/NEO-EXT trials (9.0 years and 0.5–2.2 years, respectively) and younger mean age at diagnosis (36.2 years and 44.7–48.6 years, respectively). Least squares mean (95% confidence interval) improvement from baseline in FVCpp at Week 49–52 for AVA versus ALG was 2.43 (−0.13; 4.99) for COMET (*n* = 98); 2.31 (0.06; 4.57) for Model 1 (*n* = 160); 2.43 (0.21; 4.65) for Model 2 (*n* = 157); 2.80 (0.54; 5.05) for Model 3 (*n* = 154); and 2.27 (−0.30; 4.45) for Model 4 (*n* = 101).

**Conclusions:**

Models 1 to 3, which had an increased sample size versus COMET, demonstrated a nominally significant effect on FVCpp favoring AVA versus ALG after 1 year of treatment, consistent with results from COMET.

## Highlights

• Clinical trials in Pompe disease are challenging due to small patient numbers.
• Avalglucosidase alfa (AVA) and alglucosidase alfa (ALG) trial data were pooled.
• Regression analyses compared FVCpp between AVA and ALG.
• A nominally significant effect on FVCpp favoring AVA versus ALG was demonstrated.

## 1 Introduction

Pompe disease is a rare, progressive neuromuscular disease caused by deficiency of acid α-glucosidase (GAA), an enzyme that breaks down glycogen [1]. This deficiency of GAA, caused by pathogenic variants in the *GAA* gene, results in accumulation of lysosomal glycogen, leading to cellular dysfunction, muscle damage and functional disability [1]. In contrast to infantile-onset Pompe disease (IOPD), patients with the late-onset Pompe disease (LOPD) phenotype experience a more variable disease course and rate of progression [2]. The progressive muscle damage associated with LOPD causes a decline in respiratory and motor function, associated with substantial morbidity and mortality if inadequately treated [3].

The treatment landscape for LOPD is changing; until recently, the standard-of-care has been enzyme replacement therapy (ERT) with recombinant human acid α-glucosidase (alglucosidase alfa [ALG]; Lumizyme® in the US, Myozyme® in other regions), which has been available since 2006 [[3], [4], [5]]. ERT with ALG improves walking distance, stabilizes respiratory function, and extends invasive ventilator-free survival in children and adults with LOPD [6,7]. However, limited targeting of ALG to skeletal muscle impacts its effects, and the overall disease burden in LOPD remains high despite ERT [8,9]. This has driven a need for therapies with improved efficacy and long-term effects. Avalglucosidase alfa (AVA) is a next-generation ERT with improved targeting to skeletal muscle and trafficking to lysosomes compared with ALG [10], which is approved for the treatment of patients with LOPD and/or IOPD in several countries worldwide [11,12]. Evidence on the clinical efficacy of AVA in improving respiratory function in LOPD is available from several clinical trials including a Phase 1 open-label trial (NEO1; NCT01898364) and its open-label extension (Neo-EXT; NCT02032524) [13,14], and the Phase 3 COMET trial (NCT02782741) that compared the efficacy of AVA with ALG [15]. In the COMET trial, the difference in change from baseline in upright forced vital capacity percent predicted (FVCpp) for AVA versus ALG was 2.43% (95% confidence interval [CI] -0.13; 4.99), demonstrating non-inferiority (*p* *=* 0.0074; non-inferiority margin 1.1) and achieving the study's primary objective [15]. Superiority of AVA versus ALG was narrowly missed (*p* = 0.0626), which may have been due to an outlying patient in the AVA arm with low baseline FVC and severe chronic obstructive pulmonary disease [15,16]. This highlights the challenges of clinical trials in rare diseases, in which the sample size is often small. Using pooled data from multiple studies and analyzing common endpoints provides the opportunity to increase the sample size and obtain additional insights into clinical outcomes.

Thus, to fully assess the benefit of AVA on FVCpp compared with ALG, a post hoc analysis with a larger sample size was conducted using pooled data from the COMET and NEO1/NEO-EXT trials of AVA and the LOTS trial of ALG [6].

## 2 Materials and methods

### 2.1 Data sources and patients

This post hoc analysis included pooled individual patient data from the COMET (NCT02782741), NEO1 (NCT01898364)/NEO-EXT (NCT02032524), and LOTS (NCT00158600) clinical trials, which investigated AVA and/or ALG for the treatment of LOPD and included change from baseline in upright FVCpp as an outcome. All available clinical trial data for AVA (NEO1, NEO-EXT, and COMET) and ALG (LOTS and COMET) were included in the analysis. A summary of these trials is shown in Table 1, and full details of each trial have been published previously [6,[13], [14], [15]].

**Table 1:** Trials and patients included in the analysis.

Trial	COMET (NCT02782741) [15]	LOTS (NCT00158600) [6]	NEO1 (NCT01898364) / NEO-EXT (NCT02032524) [13, 14]
Design	Phase 3, randomized, double-blind trial comparing biweekly infusions of AVA versus ALG	Phase 3, randomized, double-blind, placebo-controlled trial	Phase 1, open-label, dose-escalation trial (NEO1) and Phase 2 open-label LTE study of patients who received AVA in NEO1 (NEO-EXT)
Intervention	AVA (biweekly 20 mg/kg) or ALG (biweekly 20 mg/kg) for 49 weeks⁎	ALG (biweekly 20 mg/kg) or placebo for 78 weeks	AVA (biweekly 5, 10, or 20 mg/kg) for 24 weeks (NEO1), followed by AVA (biweekly 20 mg/kg) for up to 6.5 years†
Key inclusion criteria	• Confirmed GAA enzyme deficiency from any tissue source and/or two confirmed *GAA* gene mutations • Treatment-naïve • ≥3 years of age • Upright FVCpp 30–85% • No requirement for invasive ventilation	• Diagnosis of Pompe disease based on deficient endogenous GAA activity in cultured skin fibroblasts of ≤40% of the normal mean of the testing laboratory and two *GAA* gene mutations • Treatment-naive • ≥8 years of age • Upright FVCpp 30–<80% • No requirement for invasive ventilation	• Confirmed GAA enzyme deficiency from any tissue source and/or confirmed *GAA* gene mutation and without known cardiac hypertrophy • ERT-naive and ERT-experienced • ≥18 years of age • Upright FVCpp ≥50% • No requirement for invasive ventilation
Primary endpoint	Change from baseline to Week 49 in upright FVCpp	Upright FVCpp and 6MWT at Week 78	Safety and tolerability§
Patients considered in the current analysis‡	ERT-naïve patients randomized to receive biweekly AVA 20 mg/kg (n *=* 51) or ALG 20 mg/kg (n *=* 49)	ERT-naïve patients randomized to receive biweekly ALG 20 mg/kg (*n* = 60)	ERT-naive patients treated with biweekly AVA 20 mg/kg (n *=* 3)


Demographics and baseline characteristics of included patients				
	AVA (N *=* 51)	ALG (N *=* 49)	ALG (N *=* 60)	AVA (N *=* 3)
Sex, n (%)				
Male	27 (52.9)	25 (51.0)	34 (56.7)	2 (66.7)
Female	24 (47.1)	24 (49.0)	26 (43.4)	1 (33.3)
Race, n (%)				
White	47 (92.2)	47 (95.9)	57 (95.0)	3 (100.0)
Other	4 (7.8)	2 (4.1)	3 (5.0)	0 (0.0)
Mean (SD) age at onset of symptoms, years	32.9 (16.6)	37.7 (15.7)	30.3 (12.3)	38.3 (NA)¶
Mean (SD) duration of disease,†† years	1.3 (2.67)	2.2 (5.0)	9.0 (6.3)	0.5 (0.7)
Use of walking device,‡‡ n (%)	7 (13.7)	10 (20.4)	23 (38.0)	1 (33.3)
Use of ventilation, n (%)				
Yes	13 (25.5)	11 (22.4)	20 (33.3)	*NA*
Missing	2 (3.92)	1 (2.04)	0 (0.0)	3 (100)
Mean (SD) 6MWT performance, m	399.3 (110.9)	378.1 (116.2)	332.2 (126.7)	502.7 (125.0)
Mean (SD) FVCpp	62.6 (14.4)	61.6 (12.4)	55.4 (14.4)	63.7 (16.3)
Mean (SD) weight, kg	77.76 (22.1)	79.3 (18.2)	73.4 (17.6)	67.6 (12.6)
Mean (SD) age at diagnosis, years	44.7 (14.7)	48.2 (14.6)	36.2 (13.3)	48.6 (26.0)
Region, n (%)				
Asia-Pacific	4 (7.8)	1 (2.0)	0 (0.0)	0 (0.0)
Europe	31 (60.8)	21 (42.9)	21 (35.0)	3 (100.0)
Latin America	2 (3.9)	7 (14.3)	0 (0.0)	0 (0.0)
North America	14 (27.5)	20 (40.8)	39 (65.0)	0 (0.0)
Ethnicity, n (%)				
Hispanic or Latino	3 (5.9)	12 (24.5)	1 (1.7)	0 (0.0)
Not Hispanic or Latino	44 (86.3)	32 (65.3)	0 (0.0)	3 (100.0)
Unknown	4 (7.8)	5 (10.2)	59 (98.3)	0 (0.0)
Mean (SD) age at enrolment, years	46.0 (14.5)	50.3 (13.4)	45.3 (12.4)	49.1 (25.6)
Country, n (%)				
France	7 (13.7)	5 (10.2)	7 (11.7)	0 (0.0)
Netherlands	2 (3.9)	3 (6.1)	14 (23.3)	0 (0.0)
United States	12 (23.5)	20 (40.8)	39 (65.0)	0 (0.0)
Others	30 (58.8)	21 (57.1)	0 (0.0)	3 (100.0)

COMET primary analysis period: 2016–2020; LOTS study period: 2005–2007; NEO1 study period: 2013–2015; NEO-EXT (interim data cut-off): 2014–2020.

6MWT, 6-min walk test; ERT, enzyme replacement therapy; FVCpp; forced vital capacity percent predicted; GAA, acid α-glucosidase; LOPD, late-onset Pompe disease; LTE, long-term extension; m, meters; NA, not applicable; SD, standard deviation.

⁎ After Week 49, patients treated with alglucosidase alfa transitioned to avalglucosidase alfa.

† In NEO-EXT, patients from NEO1 continued their dose and transitioned to biweekly 20 mg/kg.

‡ In all trials, patients considered for the pooled analysis were treatment-naive with LOPD.

§ Pulmonary function endpoints were exploratory endpoints.

¶ Age at onset of symptom was only available for 1 patient in NEO1 study.

†† For COMET and LOTS disease duration was calculated as ‘age at first infusion – age at diagnosis’, for NEO1/NEO-EXT, it was calculated as ‘age at enrolment – age at diagnosis’.

‡‡ For LOTS walking device included crutch, orthotics, rolling walker, and cane; for COMET it included cane, wheelchair, corset, walker, and scooter; for NEO1/NEO-EXT it included rolling walker.

COMET was a Phase 3 randomized double-blind trial of intravenous (IV) AVA 20 mg/kg biweekly versus IV ALG 20 mg/kg biweekly in treatment-naive patients with LOPD who were ≥ 3 years of age with upright FVCpp 30–85% [15]. Data were included from the 49-week, double-blind treatment period [15]. NEO1 was a Phase 1/2, open-label, ascending-dose trial of IV AVA 5, 10, or 20 mg/kg biweekly for 6 months in treatment-naive or ERT-experienced patients ≥18 years of age with upright FVCpp ≥50% [13]. Patients from NEO1 continued their assigned dose of AVA for 104–156 weeks in the open-label extension trial NEO-EXT; all patients then receive AVA 20 mg/kg biweekly for up to 8 years [14]. Only data for treatment-naive patients who received IV AVA 20 mg/kg biweekly in NEO1/NEO-EXT were included in this pooled analysis. Finally, LOTS was the pivotal Phase 3 randomized, double-blind, placebo-controlled trial of IV ALG 20 mg/kg biweekly for 78 weeks in treatment-naive patients with LOPD who were ≥ 8 years of age with upright FVCpp 30–<80% [6]. Data were included from the ALG treatment arm only. The LOTS clinical trial was conducted approximately 20 years prior to the other trials included in this analysis, hence differences in baseline patient populations were considered in the analysis.

The trial protocols were reviewed and approved by local ethics committees or institutional review boards, and the trials were conducted in accordance with the Declaration of Helsinki and the International Council for Harmonisation guidelines for Good Clinical Practice. All patients (or their guardians) provided written informed consent prior to the trials.

### 2.2 Outcomes

Patient-level data, including demographics, baseline characteristics, and outcomes were pooled for analysis.

Spirometric assessment of respiratory function was performed in each of the original trials at scheduled visits in accordance with American Thoracic Society/European Respiratory Society guidelines. FVCpp in the upright position was calculated as a function of FVC in liters, gender, race, age, and height at time of measurement using the Global Lung Initiative (GLI) 2012 reference eqs. [17]. The LOTS trial was conducted between 2005 and 2007, before the 2012 GLI reference equations, therefore, the FVCpp values for this trial were rederived for this analysis in order to match the reference values used in the COMET trial [15].

To enable comparison across trials, assessment time points were grouped into the following intervals: Week 12–13, Week 25–26, Week 37–38 (not available for NEO1/NEO-EXT), and Week 49–52 (Supplementary Table 1).

### 2.3 Statistical analyses

#### 2.3.1 Demographics and baseline characteristics were summarized descriptively

Regression analyses were conducted to compare change from baseline in FVCpp between AVA and ALG at post-baseline scheduled assessments up to 1 year, using Stata 15 software (StataCorp. 2017. *Stata Statistical Software: Release 15*. College Station, TX: StataCorp LLC.). A restricted maximum likelihood repeated measures model (MRMM) was used with an unstructured covariance matrix to account for the dependency between the assessments from the same patient using Week 12–13, Week 25–26, Week 37–38, and Week 49–52 assessments as the dependent variable, and treatment, study (to capture any residual study effect), visit (categorical), interaction between treatment and visit, and baseline covariates to adjust for the differences between trials. The addition of covariates was based on the statistical significance; the effect of adding the covariate on the statistical goodness of fit of the model, as given by Akaike's information criteria and Bayesian information criteria [18,19]; the difference in mean value of the covariate at baseline across trials; and previously obtained clinical inputs and results from subgroup analyses from the COMET trial. Based on these criteria, the baseline variables selected for adjustment were sex, duration of disease (years), use of walking device (yes/no), use of ventilation (yes/no), 6-min walk test (6MWT, meters), FVCpp (%), weight (kg), and age (years). The following four regression models were developed: Model 1 included data from COMET, NEO1/NEO-EXT, and LOTS, and had the largest sample size (*n* *=* 160); Model 2 included data from COMET and LOTS (*n* *=* 157); Model 3 included data from COMET and LOTS, with additional adjustment for baseline ventilation use (*n* *=* 154); and Model 4 included only data on AVA, from COMET and NEO1/NEO-EXT (with a similar sample size to the COMET trial; *n* *=* 101). An exploratory analysis of Model 2 was also conducted in which three patients from COMET with missing data on baseline ventilation (AVA arm *n* *=* 2, ALG arm n = 1) were excluded. All regression models were consistent with the pre-specified COMET trial model, which also used MRMM with an unstructured covariance matrix; however, additional covariates were not included in the COMET trial model. The covariates used in COMET and each of the four models are presented in Table 2. Estimated differences in the change from baseline in FVCpp between treatments for each model were summarized as least squares (LS) means with 95% CI, calculated using the predicted standard error for the linear combination (generated via the predict statement in Stata 15).

**Table 2:** Covariates included in the models.

Covariate	COMET	Model 1 (COMET, NEO1/NEO-EXT, LOTS)	Model 2 (COMET, LOTS)	Model 3 (COMET, LOTS)	Model 4 (COMET, NEO1/NEO-EXT)
Study	NA	X	X	X	X
Treatment (avalglucosidase alfa/alglucosidase alfa)	X	X	X	X	X
Visit (Week 12–13/25–26/37–38/49–52)	X	X	X	X	X
Treatment-by-visit interaction	X	X	X	X	X
Baseline FVCpp (%)	X	X	X	X	X
Baseline 6MWT (meters)	–	X	X	X	X
Sex (male/female)	X	X	X	X	X
Baseline age (years)	X	X	X	X	X
Disease duration (years)	–	X	X	X	X
Baseline weight (kg)	–	X	X	X	X
Baseline walking device (yes/no)	–	X	X	X	X
Baseline ventilation use (yes/no)	–	–	–	X	–

‘X' represents covariates included in the model; ‘–’ represents covariates not included in the model. For all models, region and country were not included as covariates as they were not expected to be prognostic factors, race was not included as across the trials only a small number of patients were non-white, and age at symptom onset and ethnicity were not included as data were only available for one patient in the NEO1 and LOTS studies, respectively. Age at diagnosis was indirectly captured using disease duration, so was also not included as a covariate. 6MWT, 6-min walk test; FVCpp; forced vital capacity percent predicted; NA, not applicable.

## 3 Results

### 3.1 Patients

Overall, 100 randomized patients from COMET (AVA, *n* *=* 51, ALG, *n* *=* 49), 60 randomized patients from LOTS (ALG arm only), and three patients from NEO1/NEO-EXT (treatment-naive patients who received open-label AVA only) were considered for analysis.

Mean age at enrollment was similar across trials (45.3–50.3 years; Table 1). The majority of patients in all trials were white and from Europe or North America. While mean age at first symptoms was similar across trials, patients from the LOTS trial had longer mean duration of disease compared with the COMET and NEO1/NEO-EXT trials (9.0 years and 0.5–2.2 years, respectively) and younger mean age at diagnosis (36.2 years and 44.7–48.6 years, respectively; Table 1). Compared with patients from COMET or NEO1/NEO-EXT, patients enrolled in LOTS were also more likely to require walking devices (13.7–33.0% versus 38.0%, respectively) and ventilation assistance (22.4–25.5% versus 33.3%, respectively; Table 1). Baseline mean FVCpp was similar for the COMET and NEO1/NEO-EXT trials (61.6–63.7%), but numerically lower in the LOTS trial population (55.4%; Table 1).

**Fig. 1:**
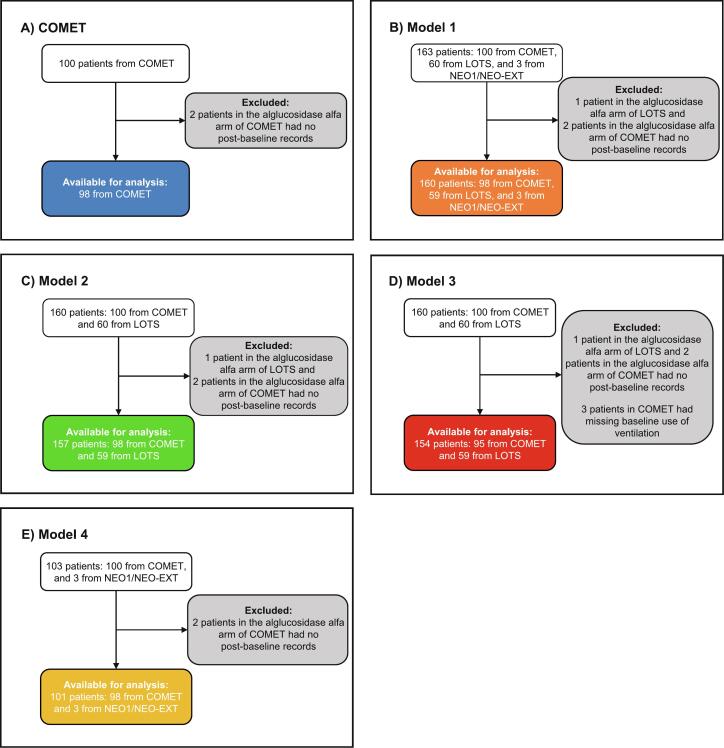
Patient disposition for COMET (A) and regression models, Model 1 (B), Model 2 (C), Model 3 (D), and Model 4.

Post-baseline data were available for 160 patients across the three clinical trials (COMET, *n* *=* 98, LOTS, *n* *=* 59, NEO1/NEO-EXT, *n* *=* 3); two patients from the ALG arm of the COMET trial and one patient from the ALG arm of the LOTS trial had no post-baseline data available and were excluded from the regression analyses. The number of post-baseline observations available at each time interval in each trial is shown in Supplementary Table 2. For each model, different study populations and covariates were used, therefore, the number of observations and patients included in these models varied; patient disposition for each model is shown in Fig. 1. Coefficient estimates for each model are presented in Supplementary Tables 3–6.

### 3.2 Regression analyses of relative effects on FVCpp

**Fig. 2:**
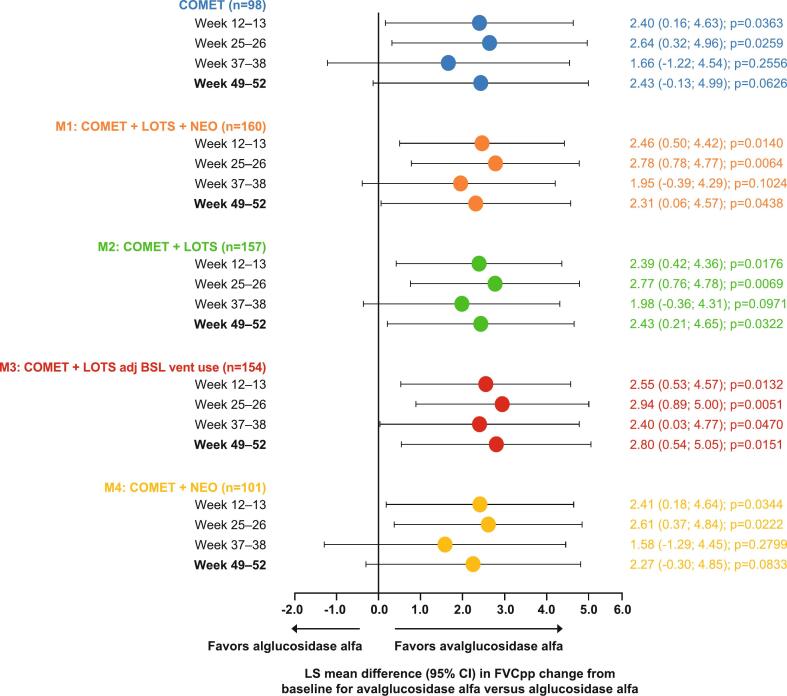
LS mean difference in FVCpp change from baseline for avalglucosidase alfa versus alglucosidase alfa. Nominal *p*-values are presented. adj BSL, adjusted for baseline ventilator use; ALG, alglucosidase alfa; AVA, avalglucosidase alfa; CI, confidence interval; FVCpp, forced vital capacity percent predicted; LS, least squares.

The relative LS mean difference in improvement in FVCpp for up to 1 year with AVA versus ALG in the COMET trial and in pooled analyses based on each of the four regression models is shown in Fig. 2. The corresponding changes from baseline for AVA and ALG are shown in Supplementary Table 7.

In the analysis based on the COMET trial (number of patients = 98; number of observations = 381), the LS mean (95% CI) difference in improvement in FVCpp with AVA versus ALG at Week 49–52 was 2.43 (−0.13; 4.99); however, superiority was not significantly different (*p* = 0.063) [15]. Improvement in FVCpp during COMET favored AVA over ALG at Weeks 12–13, 25–26, and 37–38 (Fig. 2; Supplementary Table 7), reaching nominal significance at Week 12–13 (2.40 [0.16; 4.63]) and Week 25–26 (2.64 [0.32; 4.96]).

Model 1 included data from COMET, NEO1/NEO-EXT, and LOTS (number of patients = 160; number of observations = 617). Change from baseline in FVCpp was numerically greater with AVA versus ALG at all time points (Supplementary Table 7). The LS mean (95% CI) treatment difference estimated from the model at Weeks 49–52 was 2.31 (0.06; 4.57). Nominal significance was reached at all time points except for Weeks 37–38 (Fig. 2; Supplementary Table 7).

Model 2 included data from the COMET and LOTS trials (number of patients = 157; number of observations = 608). There was a numerically greater change from baseline in FVCpp with AVA versus ALG at all time points (Supplementary Table 7). The estimated LS mean (95% CI) treatment difference at Weeks 49–52 for AVA versus ALG was 2.43 (0.21; 4.65). The relative predicted treatment effect of AVA on FVCpp was greater than that for ALG at all time points, reaching nominal significance at all weeks except of Weeks 37–38 (Fig. 2; Supplementary Table 7). These results were aligned with results reported in the COMET trial and Model 1, with the treatment difference the same value as reported in the COMET trial but nominally significant.

Model 3 included data from the COMET and LOTS trials and was adjusted for baseline ventilation use (number of patients = 154; number of observations = 596). Patients from the COMET trial with missing baseline data on ventilation use (*n* *=* 3; AVA arm *n* *=* 2, ALG arm = 1) were excluded from this model. A numerically greater change from baseline in FVCpp was observed with AVA versus ALG at all time points (Supplementary Table 7). At Weeks 49–52, the LS mean (95% CI) estimated treatment difference (2.80 [0.54; 5.05]) was nominally significant in favor of AVA (Fig. 2; Supplementary Table 7). Predicted relative effects were also nominally significantly in favor of AVA at all other time points. Adjusting for ventilation use resulted in a higher effect size estimate for AVA versus ALG compared with the other models and the COMET trial. An exploratory analysis of Model 2, which excluded the three patients with missing baseline data on ventilation use from the COMET trial, resulted in similar results to Model 3, with a LS mean (95% CI) estimated treatment difference of 2.74 (0.49; 4.99) at Weeks 49–52 (Supplementary Table 8).

Model 4 included data from the COMET and NEO1/NEO-EXT trials (number of patients = 101; number of observations = 390). LS mean change from baseline in FVCpp was consistently greater with AVA versus ALG (Supplementary Table 7), with nominally significant treatment differences predicted at Weeks 12–13 and Weeks 25–26 (Fig. 2; Supplementary Table 7). Estimated treatment effect on FVCpp was numerically greater at Weeks 37–38 (LS mean difference [95% CI] 1.58 [−1.29; 4.45]) and Weeks 49–52 (LS mean difference [95% CI] 2.27 [−0.30; 4.85]), but did not reach nominal significance.

## 4 Discussion

The objective of this post hoc analysis was to gain more insights into the relative efficacy of AVA versus ALG on FVCpp by constructing regression models of pooled data from three clinical trials to increase precision: COMET, NEO1/NEO-EXT, and LOTS. Four regression models were developed for estimation of the treatment effect on FVCpp. An effect favoring AVA versus ALG was demonstrated at approximately 1 year in all four models, with nominal significance demonstrated in three of the four models (Models 1–3, ranging from 2.27 to 2.80) where the sample size was substantially greater than in the COMET trial, with an estimated effect on FVCpp consistent with the results from the pre-specified COMET regression analyses. Nominally significant improvements with AVA versus ALG were also observed in all four models at Weeks 12–13 and 25–26, in Model 3 at Weeks 37–38, and in Models 1, 2, and 3 at Weeks 49–52. In all analyses, results numerically favored AVA over ALG at each time point, as expected based on the primary results from COMET [15].

Model 2, which included data from the double-blind randomized trials COMET and LOTS and was based on 608 observations from 157 patients, was considered as providing the most reliable estimates. Other models that included data from the NEO1/NEO-EXT trials (Models 1 and 4) were limited by the small number of treatment-naive patients included from this trial (*n* *=* 3), therefore, predicted treatment differences should be interpreted with caution. A sensitivity analysis of Model 2 was also conducted (Model 3), which was adjusted for baseline ventilation use. While this model demonstrated a greater effect size estimate than Model 2, this result may have been driven by the exclusion of patients from COMET without baseline data on ventilation use, as demonstrated by an exploratory analysis of Model 2 in which these patients were excluded and the effect size at Weeks 49–52 was in line with Model 3.

The results of our analysis are consistent with those of the COMET trial, in which a clinically meaningful improvement in respiratory function at 1 year was observed with AVA over ALG in treatment-naive patients with LOPD [15]. The relative effect estimated at this time point in COMET was 2.43% (95% CI -0.13; 4.99), which did not reach statistical significance for superiority [15]. In comparison, in Model 2 the estimated relative effect at 1 year was 2.43% (95% CI 0.21; 4.65), the same as reported in the COMET trial, but with smaller confidence intervals due to the increased sample size in the model. These results support the hypothesis that superiority of AVA over ALG in the original COMET analysis was likely missed due to insufficient sample size in the presence of an outlying patient [16]. Additional post-hoc non-parametric analyses of FVC in the COMET trial examined the statistical impact of this outlier with low baseline FVC and severe chronic obstructive pulmonary disease, and showed a significant *p*-value based on all observed data (*p* = 0.019) [16]. The benefits on upright FVC are of clinical significance because FVC is predictive of respiratory failure and the requirement for invasive ventilation [20], as well as patient-reported outcomes including health-related quality of life [21]. Given these points, as well as the progressive nature of LOPD, even stability in respiratory function can be clinically meaningful.

Clinical and real-world studies of ALG have shown stabilization or improvements in respiratory function and survival demonstrated over several years [[22], [23], [24]]. However, recent data from an observational study of patients receiving ALG with a mean (standard deviation [SD]) follow-up of 7 (3) years demonstrated that initial improvement in FVCpp was followed by a statistically significant deterioration, particularly in those patients with more severe disease at baseline [25]. Longer-term data on the real-world effectiveness of AVA in LOPD are not yet available, and the potential clinical benefits of enhanced cellular uptake of AVA in skeletal muscle on maintenance of effect requires further investigation. Data from the open-label, extended treatment period of the COMET trial (final data cut-off 31st May 2023) demonstrated that respiratory and motor function were stabilized or improved at Week 145 from baseline, demonstrating the continued benefit of AVA beyond the primary analysis period [26]. Additionally, available data from an interim analysis of the NEO-EXT trial show stabilization of upright FVCpp over up to 6 years of treatment with AVA in a sample of 10 treatment-naive patients and 14 patients who switched from ALG after ≥9 months treatment [14]. These results inform our understanding of the longer-term efficacy and safety of AVA in patients with LOPD.

Limitations of this analysis include its post hoc nature and the use of pooled data from trials with different designs and different inclusion criteria. For example, included patients from the NEO1/NEO-EXT trials were restricted to those considered to be most comparable with patients from COMET, therefore, study was included as a fixed effect in all regression models in order to capture the residual study effect. All models favored AVA, with significant treatment differences at 1 year in Models 1–3; however, statistical significance was nominal (i.e., exploratory) as these analyses were not pre-specified in the original plan from the trials and were not adjusted for multiple testing. Results that included data from the NEO1/NEO-EXT trials must also be interpreted with caution due to the small number of patients included from these trials. In addition, changes in the standard-of-care for patients with LOPD since the LOTS trial was conducted may have influenced responses in patients treated with AVA in COMET. Finally, no imputation was performed for missing data; however, the proportion of missing covariate data was low in a trial setting and the repeated measures modeling addressed missing outcome data without the need for imputation. Despite these limitations, these pooled data from different clinical trials provide valuable information on the treatment of LOPD, a rare disease with a heterogenous presentation and disease course in which clinical trials are often hampered by small sample sizes. The advent of artificial intelligence may help address some of these issues by aiding in earlier diagnosis and identification of patients [27]. In the absence of such advances, pooled analyses such as the current analysis can be informative in understanding the value of new medications for rare diseases.

In conclusion, results from this pooled analysis of trial data that increases the sample size reinforce the suggested superiority of AVA over ALG on FVCpp. Overall, these data demonstrate that AVA is associated with a more favorable effect on respiratory function at 1 year than ALG in treatment-naive patients with LOPD.

## Funding

This analysis was sponsored by 10.13039/100004339Sanofi.

## CRediT authorship contribution statement

**Tahseen Mozaffar:** Writing – review & editing, Validation, Investigation. **Lionel Riou França:** Writing – review & editing, Writing – original draft, Validation, Methodology, Conceptualization. **Jérôme Msihid:** Writing – review & editing, Writing – original draft, Validation, Methodology, Data curation, Conceptualization. **Pragya Shukla:** Writing – review & editing, Writing – original draft, Validation, Methodology, Formal analysis, Conceptualization. **Irina Proskorovsky:** Writing – review & editing, Validation, Methodology, Formal analysis, Conceptualization. **Tianyue Zhou:** Writing – review & editing, Writing – original draft, Validation, Conceptualization. **Magali Periquet:** Writing – review & editing, Writing – original draft, Validation, Conceptualization. **Kristina An Haack:** Writing – review & editing, Writing – original draft, Validation, Conceptualization. **Laurence Pollissard:** Writing – review & editing, Writing – original draft, Validation, Supervision, Project administration, Conceptualization. **Volker Straub:** Writing – review & editing, Validation, Investigation.

## Declaration of competing interest

TM has participated in advisory boards for AbbVie, Alexion, Amicus, Argenx, Audentes, Maze Therapeutics, Momenta, Ra-Pharmaceuticals, Sanofi, Sarepta Therapeutics, Spark Therapeutics, UCB, Ultragenyx, and Zogenix, and Speaker's bureau for Sanofi; and has received research funding from Alexion, Amicus, Argenx, Audentes, Bristol Myers-Squibb, Cartesian, Grifols, Momenta, Ra-Pharmaceuticals, Sanofi, Spark Therapeutics, UCB, and Valerion.

JM, KAH, TZ, and LP are employees and may hold stock and/or stock options in Sanofi.

MP: was an employee of Sanofi at the time of study participation and may still hold stock and/or stock options, and is currently employed as the Global Medical Lead for Early Indications and New Programs – Rare Diseases at UCB.

LRF was an employee of Sanofi at the time the research took place and is now an employee of Aixial, a contract research organization working with Sanofi. He holds stock in Sanofi.

PS and IP are employees of Evidera, part of ThermoFisher Scientific.

VS has received consulting fees from Novartis Gene Therapies, Roche, Sanofi, Sarepta Therapeutics, and Vertex Pharmaceuticals, and honoraria from Sanofi; and has conducted contracted research for Sanofi and Sarepta Therapeutics.

## Data Availability

Data will be made available on request.

## Appendix A Supplementary data

Supplementary Tables 1 to 7. Image 1
