# Supplementary material for: Efficacy of avalglucosidase alfa on forced vital capacity percent predicted in treatment-naïve patients with late-onset Pompe disease: A pooled analysis of clinical trials
Source: Mol Genet Metab Rep. 2024 Jun 26;40:101109. doi: 10.1016/j.ymgmr.2024.101109 (PMC11259910; doi:10.1016/j.ymgmr.2024.101109)
Supplement: Supplementary file 1 — Supplementary Tables 1 to 7. [file mmc1.pdf]

Efficacy of avalglucosidase alfa on respiratory function in treatment-naïve patients with late-onset Pompe disease: a pooled analysis of clinical trials

Tahseen Mozaffar, Lionel Riou França, Jérôme Msihid, Pragya Shukla, Irina Proskorovsky, Tianyue Zhou, Magali Periquet, Kristina An Haack, Laurence Pollissard, Volker Straub

Supplementary material

Supplementary Table 1. Time points used for comparison across trials

**Time point**	**COMET**	**LOTS**	**NEO1/NEO-Ext**
**Week 12–13**	Week 13	Week 12	Week 13
**Week 25–26**	Week 25	Week 26	Week 25
**Week 37–38**	Week 37	Week 38	-
**Week 49–52**	Week 49	Week 52	Week 52

Supplementary Table 2. Number of post-baseline observations available for regression analyses of change from baseline in FVCpp

**Time point**	**Avalglucosidase alfa observations, n**			**Alglucosidase alfa observations, n**		
	**COMET**	**NEO1/NEO-EXT**	**Total**	**COMET**	**LOTS**	**Total**
**Week 12–13**	51	3	54	47	58	105
**Week 25–26**	51	3	54	45	57	102
**Week 37–38**	51	0	51	44	55	99
**Week 49–52**	49	3	52	43	57	100

n= number of observations. FVCpp, forced vital capacity percent predicted

Supplementary Table 3. Model 1 coefficient estimates

Variables	Coefficient estimate	SE	95% CI	P-value
**Intercept**	0.614	3.225	-5.758; 6.986	0.849
**Treatment: AVA (reference: ALG)**	2.460	1.012	0.461; 4.458	0.016
**Study: (reference: COMET)**				
**LOTS**	1.245	1.095	-0.919; 3.409	0.258
**NEO1/NEO-EXT**	3.013	2.871	-2.660; 8.687	0.296
**Baseline FVCpp (%)**	0.032	0.030	-0.028; 0.092	0.294
**Visit (reference: Week 12–13)**				
Week 25–26	-0.213	0.407	-1.016; 0.590	0.601
Week 37–38	-0.419	0.469	-1.345; 0.508	0.373
Week 49-52	-0.192	0.476	-1.132; 0.748	0.687
**Interaction between visit and treatment (AVA)**				
Week 25–26	0.318	0.691	-1.048; 1.683	0.647
Week 37–38	-0.510	0.801	-2.092; 1.073	0.526
Week 49–52	-0.145	0.811	-1.748; 1.458	0.859
**Sex: Female (reference: male)**	1.330	0.895	-0.439; 3.098	0.139
**Age at enrollment (years)**	-0.024	0.030	-0.084; 0.035	0.419
**Disease duration (years)**	0.031	0.080	-0.127; 0.189	0.697
**Use of walking device at baseline (reference: No walking device)**	-0.068	1.076	-2.194; 2.057	0.950
**Baseline 6MWT (meters)**	0.002	0.004	-0.006; 0.010	0.638
**Baseline weight (kg)**	-0.029	0.021	-0.071; 0.013	0.177

6MWT, 6-minute walk test; AVA, avalglucosidase alfa; ALG, alglucosidase alfa; CI, confidence interval; FVCpp, forced vital capacity percent predicted; SE, standard error

Supplementary Table 4. Model 2 coefficient estimates

Variables	Coefficient estimate	SE	95% CI	P-value
**Intercept**	1.054	3.263	-5.393; 7.501	0.747
**Treatment: AVA (reference: ALG)**	2.388	1.017	0.380; 4.397	0.020
**Study: LOTS (reference: COMET)**	1.210	1.099	-0.963; 3.382	0.273
**Baseline FVCpp (%)**	0.032	0.030	-0.028; 0.092	0.294
**Visit (reference: Week 12–13)**				
Week 25–26	-0.216	0.410	-1.026; 0.594	0.599
Week 37–38	-0.422	0.464	-1.338; 0.494	0.364
Week 49–52	-0.194	0.452	-1.087; 0.699	0.668
**Interaction between visit and treatment (AVA)**				
Week 25–26	0.382	0.710	-1.021; 1.785	0.591
Week 37–38	-0.412	0.798	-1.988; 1.164	0.606
Week 49–52	0.038	0.786	-1.515; 1.590	0.962
**Sex: Female (reference: male)**	1.315	0.903	-0.469; 3.100	0.147
**Age at enrollment (years)**	-0.030	0.031	-0.091; 0.030	0.326
**Disease duration (years)**	0.034	0.080	-0.125; 0.192	0.674
**Use of walking device at baseline (reference: No walking device)**	-0.168	1.085	-2.312; 1.976	0.877
**Baseline 6MWT (meters)**	0.002	0.004	-0.006; 0.010	0.647
**Baseline weight (kg)**	-0.030	0.022	-0.073; 0.012	0.163

6MWT, 6-minute walk test; AVA, avalglucosidase alfa; ALG, alglucosidase alfa; CI, confidence interval; FVCpp, forced vital capacity percent predicted; SE, standard error

Supplementary Table 5. Model 3 coefficient estimates

Variables	Coefficient estimate	SE	95% CI	P-value
**Intercept**	1.369	3.412	-5.374; 8.112	0.689
**Treatment: AVA (reference: ALG)**	2.550	1.041	0.494; 4.606	0.015
**Study: LOTS (reference: COMET)**	1.317	1.116	-0.888; 3.522	0.240
**Baseline FVCpp (%)**	0.019	0.035	-0.050; 0.087	0.594
**Visit (reference: Week 12–13)**				
Week 25–26	-0.212	0.416	-1.033; 0.609	0.611
Week 37–38	-0.500	0.463	-1.414; 0.414	0.281
Week 49–52	-0.293	0.451	-1.184; 0.598	0.517
**Interaction between visit and treatment (AVA)**				
Week 25–26	0.392	0.727	-1.045; 1.829	0.591
Week 37–38	-0.151	0.803	-1.739; 1.437	0.851
Week 49–52	0.248	0.792	-1.318; 1.814	0.755
**Sex: Female (reference: male)**	1.197	0.933	-0.646; 3.041	0.201
**Age at enrollment (years)**	-0.017	0.033	-0.081; 0.048	0.610
**Disease duration (years)**	0.030	0.081	-0.130; 0.191	0.709
**Use of walking device at baseline (reference: No walking device)**	-0.161	1.094	-2.324; 2.001	0.883
**Use of ventilation at baseline (reference: No ventilation)**	0.003	0.004	-0.006; 0.011	0.512
**Baseline 6MWT (meters)**	-0.958	1.061	-3.054; 1.138	0.368
**Baseline weight (kg)**	-0.033	0.022	-0.076; 0.010	0.137

6MWT, 6-minute walk test; AVA, avalglucosidase alfa; ALG, alglucosidase alfa; CI, confidence interval; FVCpp, forced vital capacity percent predicted; SE, standard error

Supplementary Table 6. Model 4 coefficient estimates

Variables	Coefficient estimate	SE	95% CI	P-value
**Intercept**	4.354	4.803	-5.187; 13.894	0.367
**Treatment: AVA (reference: ALG)**	2.411	1.151	0.125; 4.696	0.039
**Study: NEO1/NEOEXT (reference: COMET)**	2.857	3.205	-3.510; 9.223	0.375
**Baseline FVCpp (%)**	0.031	0.042	-0.053; 0.116	0.465
**Visit (reference: Week 12-13)**				
Week 25-26	-0.092	0.653	-1.387; 1.204	0.889
Week 37-38	-0.100	0.754	-1.597; 1.397	0.894
Week 49-52	-0.208	0.764	-1.725; 1.309	0.786
**Interaction between visit and treatment (AVA)**				
Week 25-26	0.196	0.886	-1.562; 1.955	0.825
Week 37-38	-0.831	1.028	-2.870; 1.209	0.421
Week 49-52	-0.136	1.035	-2.191; 1.919	0.896
**Sex: Female (reference: Male)**	1.403	1.264	-1.107; 3.914	0.270
**Age at enrollment (years)**	-0.046	0.041	-0.127; 0.036	0.268
**Disease duration (years)**	0.030	0.134	-0.235; 0.296	0.821
**Use of walking device at baseline (reference: No walking device)**	-0.058	1.597	-3.230; 3.114	0.971
**Baseline 6MWT (meters)**	0.000	0.006	-0.011; 0.011	0.960
**Baseline weight (kg)**	-0.055	0.029	-0.112; 0.002	0.058

6MWT, 6-minute walk test; AVA, avalglucosidase alfa; ALG, alglucosidase alfa; CI, confidence interval; FVCpp, forced vital capacity percent predicted; SE, standard error

Supplementary Table 7. Estimated improvements in FVCpp with avalglucosidase alfa versus alglucosidase alfa using regression models from the COMET trial and the present analysis (Models 1–4)

Time point	LS mean (SE) change from baseline		LS mean (SE) treatment difference (95% CI) Avalglucosidase alfa versus alglucosidase alfa	Nominal p-value
	Avalglucosidase alfa	Alglucosidase alfa		
**COMET†**				
Week 12–13	3.05 (0.78)	0.65 (0.81)	2.40 (1.13) (0.16; 4.63)*	0.0363
Week 25–26	3.21 (0.80)	0.57 (0.84)	2.64 (1.17) (0.32; 4.96)*	0.0259
Week 37–38	2.21 (1.00)	0.55 (1.05)	1.66 (1.45) (-1.22; 4.54)	0.2556
Week 49–52	2.89 (0.88)	0.46 (0.93)	2.43 (1.29) (-0.13; 4.99)	0.0626
**Model 1‡**				
Week 12–13	3.07 (0.71)	0.61 (0.71)	2.46 (1.00) (0.50; 4.42)*	0.0140
Week 25–26	3.17 (0.72)	0.39 (0.72)	2.78 (1.02) (0.78; 4.77)*	0.0064
Week 37–38	2.14 (0.88)	0.19 (80.0)	1.95 (1.19) (-0.39; 4.29)	0.1024
Week 49–52	2.73 (0.84)	0.41 (0.78)	2.31 (1.15) (0.06; 4.57)*	0.0438
**Model 2§**				
Week 12–13	3.01 (0.71)	0.62 (0.71)	2.39 (1.01) (0.42; 4.36)*	0.0176
Week 25–26	3.17 (0.73)	0.40 (0.72)	2.77 (1.03) (0.76; 4.78)*	0.0069
Week 37–38	2.17 (0.88)	0.20 (0.80)	1.98 (1.19) (-0.36; 4.31)	0.0971
Week 49–52	2.85 (0.83)	0.43 (0.77)	2.43 (91.13) (0.21; 4.65)*	0.0322
**Model 3¶**				
Week 12–13	3.18 (0.73)	0.63 (0.72)	2.55 (1.03) (0.53; 4.57)*	0.0132
Week 25–26	3.36 (0.75)	0.41 (0.73)	2.94 (1.05) (0.89; 5.00)*	0.0051
Week 37–38	2.52 (0.90)	0.13 (0.81)	2.40 (1.21) (0.03; 4.77)*	0.0470
Week 49–52	3.13 (0.85)	0.33 (0.78)	2.80 (1.15) (0.54; 5.05)*	0.0151
**Model 4ǁ**				
Week 12–13	3.03 (0.79)	0.62 (0.82)	2.41 (1.14) (0.18; 4.64)*	0.0344
Week 25–26	3.13 (0.78)	0.52 (0.83)	2.61 (1.14) (0.37; 4.84)*	0.0222
Week 37–38	2.10 (1.01)	0.52 (1.06)	1.58 (1.46) (-1.29; 4.45)	0.2799
Week 49–52	2.68 (0.90)	0.41 (0.96)	2.27 (1.31) (-0.30; 4.85)	0.0833

*95% CI does not include 0, indicating significance for nominal p-values.

†n patients=98; n observations=381; ‡n patients=160; n observations=617; §n patients=157; n observations=608;

¶Adjusted for baseline ventilation use; n patients=155; n observations=596; ǁn patients=101; n observations=390.

CI, confidence interval; FVCpp, forced vital capacity percent predicted; LS, least squares; SE, standard error

Supplementary Table 8. Exploratory analysis of Model 2: estimated improvements in FVCpp with avalglucosidase alfa versus alglucosidase alfa excluding patients with missing baseline ventilation use from the LOTS and COMET trials

**Time point**	**LS mean (SE) change from baseline**		**LS mean (SE) treatment difference between avalglucosidase alfa and alglucosidase alfa (95% CI)**
	**Avalglucosidase alfa**	**Alglucosidase alfa**	
Week 12–13	3.13 (0.73)	0.64 (0.72)	2.49 (1.03) (0.48; 4.51)
Week 25–26	3.13 (0.75)	0.43 (0.73)	2.88 (1.05) (0.83; 4.93)
Week 37–38	2.48 (0.90)	0.14 (0.81)	2.34 (1.21) (-0.03; 4.71)
Week 49–52	3.09 (0.84)	0.35 (0.78)	2.74 (1.15) (0.49; 4.99)

CI, confidence interval; FVCpp, forced vital capacity percent predicted; LS, least squares; SE, standard error
